# Application of Boundary Layer Displacement Thickness in Wind Erosion Protection Evaluation: Case Study of a *Salix psammophila* Sand Barrier

**DOI:** 10.3390/ijerph16040592

**Published:** 2019-02-18

**Authors:** Shuai Zhang, Guo-Dong Ding, Ming-han Yu, Guang-lei Gao, Yuan-yuan Zhao, Long Wang, Yi-zhao Wang

**Affiliations:** 1 Yanchi Research Station, School of Soil and Water Conservation, Beijing Forestry University, (No.) 35 Qinghua East Road, Haidian District, Beijing 100083, China; xxwoshizsxx@163.com (S.Z.); ymh_2012tai@163.com (M.-h.Y.); gaoguanglei@bjfu.edu.cn (G.-l.G.); yuanyuan0402@126.com (Y.-y.Z.); along@bjfu.edu.cn (L.W.); yizhao_wang@163.com (Y.-z.W.); 2 Key Laboratory of State Forestry Administration on Soil and Water Conservation, Beijing Forestry University, Beijing 100083, China

**Keywords:** *Salix psammophila* sand barrier, roughness, boundary layer displacement thickness, wind speed acceleration rate, effective protection area

## Abstract

Since the establishment of blown sand physics, surface roughness has been widely used in current research to indicate the ability of a surface to resist wind erosion and to evaluate the windproof effect of protective measures. However, since the calculation of surface roughness can result in different values and its applicability is poor, there are disadvantages to its use. Therefore, it is proposed that the boundary layer displacement thickness should be used rather than roughness as an indicator to solve such problems. To analyze the new indicator’s accuracy and applicability when evaluating the effect of protective measures, a wind tunnel simulation experiment on a typical mechanical protection measure commonly used for sand control in China was conducted. Indicators of roughness and boundary layer displacement thickness were compared in evaluating the windproof performance of a *Salix psammophila* sand barrier of differing heights, side lengths, and porosities. The wind speed acceleration rate and effective protection area, which can directly reflect the protective effect of a sand barrier, were analyzed as evaluation criteria. The results show that roughness can only reflect the influence of height on the windbreak effect of sand barriers, whereas the boundary layer displacement thickness accurately showed the influence of height, side length, and porosity on the windproof effect of the sand barriers. Compared with roughness, the boundary layer displacement thickness was more strongly correlated with the effective protection area. Therefore, the boundary layer displacement thickness, rather than roughness, should be used as a new indicator when evaluating the windproof effect of protective measures.

## 1. Introduction

The interaction between airflow and the surface is crucial for understanding the generation of wind erosion and the transport of sand [1,2]. Existing research uses many indicators, such as the friction coefficient, the drag coefficient, and surface roughness, to characterize the influence of surface roughness on airflow through field observations and wind tunnel simulations [3,4,5]. Among them, surface roughness is the parameter most widely used to characterize the aerodynamic characteristics of various underlying surfaces since it is highly sensitive to differences in the fundamental properties of such surfaces [6,7,8].

Surface roughness refers to the height at which the surface layer wind profile goes to zero [9]. It is a measure of the decreasing effect of the surface on the wind speed, which affects absolute and relative sediment transport by influencing the threshold wind velocity [10,11,12]. Surfaces that exhibit high resistance to wind erosion display large surface roughness values [13,14,15]. The concept of surface roughness was derived from the boundary layer theory in hydraulics research [16]. Experiments conducted by Darcy in 1854 involving 21 circular tubes with different diameters and materials showed an important phenomenon such as the friction coefficient that varied with the Reynolds number and the relative roughness of the tube wall [16]. Bagnold used the friction coefficient in reference to aerodynamics and converted it to friction velocity [17]. He was the first to propose the concept of roughness, defining it in terms of the relationship among friction flow velocity, roughness, and wind speed, and offering a method to calculate it [9]. Through wind tunnel simulation experiments, Bagnold also found that roughness is about 1/30 the average height of the surface when there is no sand movement, which is a relationship that can be used to easily calculate different underlying surfaces [17]. Since then, roughness has been widely used to indicate the anti-wind erosion ability of a surface and, thus, the effect of protective measures [18,19,20]. However, it is accepted that caution should be applied when using surface roughness for these applications. Dong proposed that the current calculation method is only an estimate of roughness and needs further improvement [21]. Ding pointed out that it is only accurate to measure the roughness of a fixed surface when the atmospheric stratification is neutral [22]. Blumberg and Greeley pointed out that, although roughness is employed by many researchers, little research has been conducted to improve our understanding of it [23]. Due to the small scale of roughness, existing technologies cannot directly measure its exact value [24,25]. The current calculation method includes measurements of the wind speed at any two heights. However, different height combinations give different results.

The boundary layer refers to a fluid layer that is close to the surface and has a non-negligible air viscous force [26]. According to aerodynamics, airflow is affected by the rough surface of the earth. The air itself is sticky and the air at the near surface is blocked. Therefore, the wind speed is reduced [27]. The air layer above the surface at which the wind speed returns to its ideal state is the boundary layer [28]. The boundary layer displacement thickness, which can be obtained by integrating the wind speed profile, refers to the thickness of the viscous fluid in the boundary layer, which must be expanded due to the decrease in flow velocity [29]. It represents the energy consumption of the air flow caused by the surface, with large values indicating greater consumption of aerodynamic energy and, therefore, a more protective effect.

**Figure 1:**
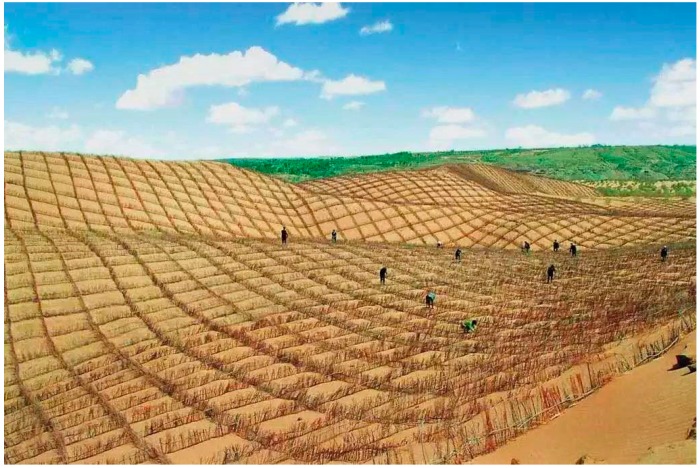
*Salix Psammophila* sand barrier.

Since there is no uniform standard for the appropriate height at which to calculate roughness, it is difficult to compare the conclusions derived from the evaluation of roughness in different studies. The boundary layer displacement thickness is obtained by integrating the wind speed profile under certain surface conditions and, because the wind speed at multiple heights can only fit one function curve, a single consistent result is calculated. The method for determining the boundary layer displacement thickness is also more accurate than that for roughness. Therefore, results from different studies can be compared and analyzed. However, at present, the application of the boundary layer displacement thickness in sandy land treatment and wind erosion protection is low, and experimental data to compare the evaluation effects of roughness and the boundary layer displacement thickness are lacking. Therefore, this study investigated a *Salix psammophila* sand barrier (Figure 1), which is a typical mechanical protection measure in the northern sandy area of China [30,31], by using wind tunnel simulation tests. The wind speed acceleration rate and effective protection area, which can directly reflect the protective effect of sand barriers, were used as the measurement indices. The accuracy of the surface roughness and boundary layer displacement thickness calculations in evaluating the protective effect of the *Salix psammophila* sand barrier was assessed, and different edge lengths, heights, and porosity were compared. Therefore, this demonstrated the applicability of boundary layer displacement thickness for evaluating the windproof effect.

## 2. Materials and Methods

### 2.1. Wind Tunnel Simulation Test

**Figure 2:**
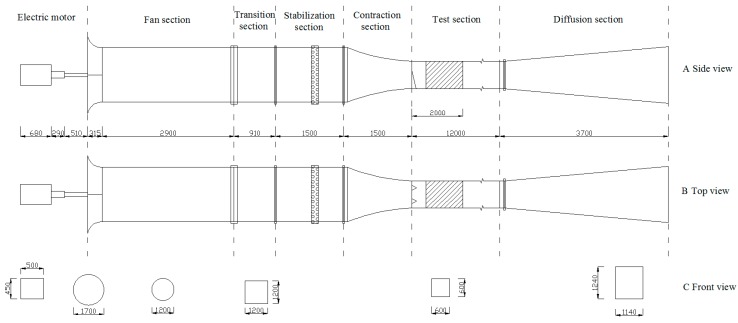
Aerodynamic outline of the wind tunnel (unit: mm).

Experiments were carried out in the wind tunnel at Mount Jiu Sand Physics Laboratory, School of Soil and Water Conservation, Beijing Forestry University. The blow-type non-circulating wind tunnel (Figure 2) has a total length of 24 m, and is composed of a power section (1.5 m long), a fan section (2.9 m long), a transition section (0.91 m long), a stabilization section (1.5 m long), a contraction section (1.5 m long), a test section (12 m long), and a diffusion section (3.7 m long). The cross section changes from circular to square at the junction of the fan section and the transition section. There is a honeycomb network at the shadow position of the stabilization section to reduce large scale eddies, and helps to fit the wind profile. The cross-sectional area of the experiment section is 0.6 × 0.6 m, and the wind speed range is continuously adjustable from 3 to 40 m s^−1^. 

We generated a scaled atmospheric boundary layer flow by using spire and roughness elements (3 cm tall, 4 cm wide, and 5 cm long). The spire (the triangle in the test section of Figure 2) has a height of 40 cm and a bottom width of 3 cm [32]. Two spires are placed and the cross section is divided into three equal parts. The roughness elements (shaded area in the test section of Figure 2) were placed within 2 m of the spire. The thickness of the boundary layer in the experiment section can reach 0.2 m. In this study, we set the wind speed gradient to 5, 8, and 11 m/s, and calculate the Reynolds number, according to the following formula [32].

Re=u∗dv

where *u* is the flow velocity, *d* is the equivalent diameter of the conduit, and *v* is the kinematic viscosity of air, *v* = 1.5 × 10^−5^ m^2^/s. We calculate the Reynolds number under different wind speed conditions as 2 × 10^5^, 3.2 × 10^5^, and 4.4 × 10^5^.

The three-dimensional displacement measurement system can adjust the position of the measuring equipment in the wind tunnel with a moving accuracy of 1 mm. The measuring equipment included a hot film anemometer (IFA300, TSI, Shoreview, MN, USA) and a hotline anemometer (KIMO, Montpon Ménestérol, France). According to previous research and the actual deployment situation in the field, the sand barrier heights were set to 0.1, 0.2, and 0.3 m, the side lengths were set to 1, 2, and 3 m, and the porosities (Longitudinal cross-sectional area ratio) to 0.2, 0.3, 0.4, and 0.5. The scaling ratio in the wind tunnel test is 1:10. Therefore, we made the model’s height and side length 10 times smaller when we made the model.

### 2.2. Wind Speed Acceleration Rate

**Figure 3:**
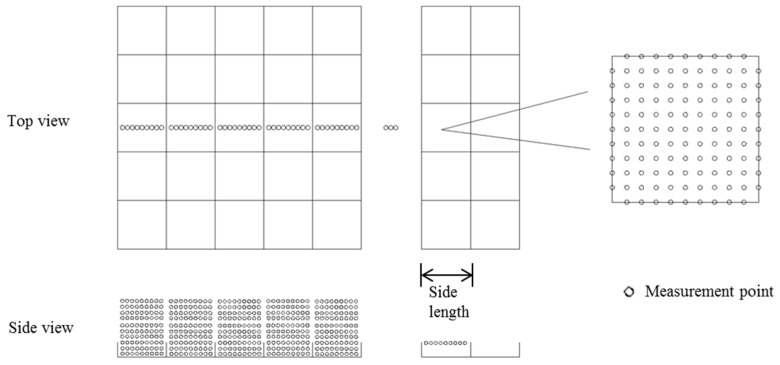
Map of wind speed flow field measurement points.

Wind speed provides a dynamic basis for wind erosion disasters and directly affects the intensity of wind erosion. The purpose of protective measures is to reduce near-surface wind speed. Therefore, it is possible to directly understand the effect of protective measures through wind speed values. The measurement points of the wind speed flow field in this study are shown in Figure 3. Wind velocity was measured from the first grid midline when testing sand barriers of different specifications. The measuring point interval was 1/10 of the side length where the lowest measuring point height was 1 cm, the highest measuring point was at 10 cm, and the height difference between adjacent measuring points of different heights was 1 cm. Measurements were taken at different grids along the wind direction until the wind speed tended to be stable and then control points were set in the next grid. Each row of control points had 9 points in each column, totaling 81 points. The distance between the control points was 1/10 of the side length, and the height was consistent with the height of the sand barrier. The control point wind speed acceleration rate was numerically simulated using Surfer (ver. 12.0, Golden Software, Llc., Golden, CO, USA), and the interpolation was performed using a Kriging method.

To understand the comparison between the wind speed without sand barrier and wind speed in the sand barrier grid, and to visually show the acceleration and deceleration zones, the following formula was used to calculate the wind speed acceleration rate.

$$a_{ij}=\frac{v_{ij}}{V_{ij}}$$

where *a_ij_* is the wind speed acceleration rate of control point at coordinates (*i, j*), *v_ij_* is the measured wind speed of the control point at coordinates (*i, j*), and *V_ij_* is the wind speed without a sand barrier of the control point at coordinates (*i*, *j*).

### 2.3. Effective Protection Area

Sand barriers can reduce near-surface wind speed and prevent sand particles from being transported and blown away. In this study, an effective protection area was used to analyze the sand-fixing capacity of sand barriers. Existing research shows that the threshold wind velocity (the height is 2 m) in bare sand is 5.0 m/s. Therefore, the area with wind speed <5.0 m/s was defined as the effective protection area. The effective protection area ratio was calculated using the following formula.

$$a=\frac{\Delta s}{S}$$

where a is the effective protection area ratio, Δ*s* is the effective protection area, and *S* is the sand barrier grid area.

### 2.4. Roughness

Bagnold proposed the following equation for calculating the wind speed distribution with elevation [17].

$$u=5.75\times u_{*}\times lg\frac{z}{z_{0}}$$

where u is wind speed, u_*_ is friction velocity, *z* is height, and *z_0_* is roughness.

The formula for the mathematical explanation of roughness is also reasonable since when height *z* = k, then lg z/k = 0, and the wind u = 0, i.e., when the height equals roughness, the wind speed is 0. Using the above formula, it can be deduced that the roughness of the underlying surface can be calculated by measuring the wind speed at any two heights.

$$\mathrm{lg}Z_{0}=\frac{\mathrm{lg}Z_{2}-A\mathrm{lg}Z_{1}}{1-A}$$

$$A=\frac{V_{2}}{V_{1}}$$

where *V_1_* is the speed at height *Z_1_, V_2_* is the speed at height *Z_2_*, and *Z_0_* is the roughness.

### 2.5. Boundary Layer Displacement Thickness

**Figure 4:**
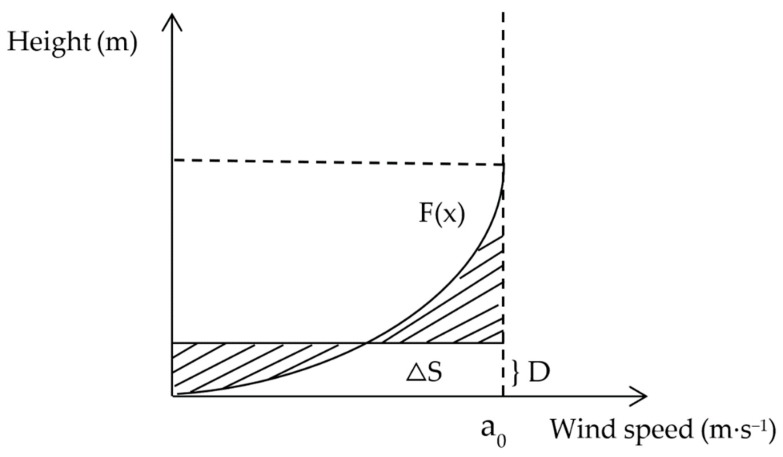
The displacement thickness of the boundary layer. D is the boundary layer displacement thickness, a_0_ is the wind speed value of airflow under ideal conditions, and F(x) is the wind profile.

The wind speed of the near-surface airflow increases with height. The curve of variation in the vertical direction is called the wind profile, which is usually fitted to an exponential or logarithmic function. The displacement thickness of the boundary layer can be understood as the thickness of all viscous fluids, which should be widened than that of non-viscous fluids, and it can be calculated by integrating the wind speed profile, as shown in Figure 4.

The mathematical meaning of the integral represents the area between the function and the wind speed (x-axis). A height (y-axis) can be found where the areas of the left and right shadows in Figure 3 are equal. The area obtained by integrating the wind speed profile is equivalent to the area of the rectangle represented by ΔS. The following formula can express the boundary layer displacement thickness.

$$D=\frac{\int_{0}^{a_{0}}f\left(x\right)dx}{a_{0}}$$

### 2.6. Statistical Analysis

One-way analysis of variance (ANOVA) and Tukey’s honestly significant difference (HSD) post hoc test were used to examine differences in roughness and boundary layer displacement thickness. Spearman’s correlation was used to test the relationships among boundary layer displacement thickness, roughness, and effective protection area. Statistical significance was accepted at *p* < 0.05. All statistical analyses were performed using SPSS software (ver. 19.0, SPSS, Inc., Chicago, IL, USA).

## 3. Results

### 3.1. Wind Speed Acceleration Rate

**Figure 5:**
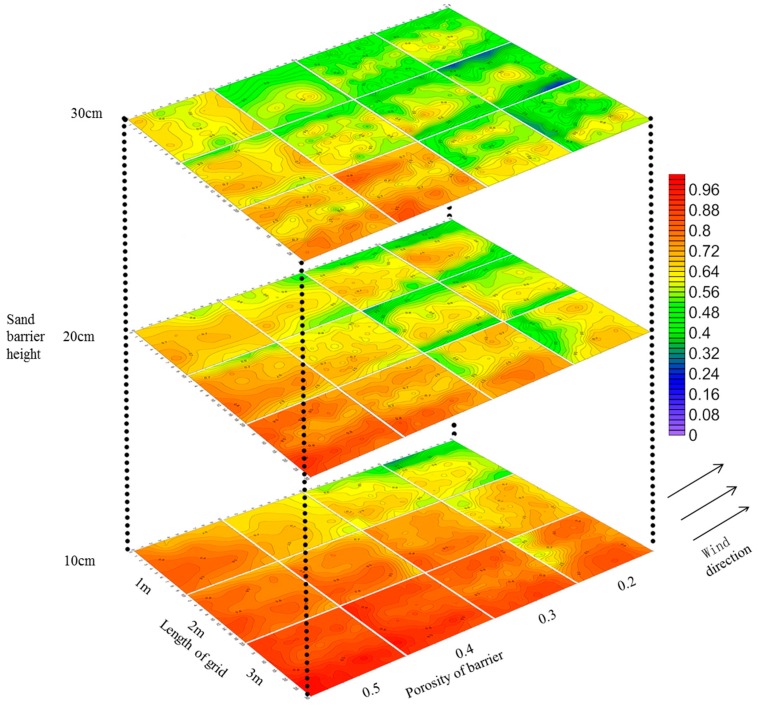
Wind acceleration rate flow field map of sand barrier with different configuration modes.

Simulations of the wind speed acceleration rate for the different configurations of the Salix psammophila sand barrier grid are shown in Figure 5. The wind speed acceleration rate was shown to be less than one for the various specifications of the Salix psammophila sand barrier. Assessment of the influence of height on the sand barrier showed that the wind speed acceleration rate decreased with increased height. When the height of the sand barrier was 10 cm, the wind speed acceleration rate was 0.7 to 0.9, and only when the side length was 1 m did it decrease to 0.5–0.7. When the height of the barrier was increased to 20 cm, the wind speed acceleration rate decreased to 0.5–0.7. However, there were still some areas where the wind speed acceleration rate reached 0.7–0.9 when the side length was 3 m. When the height of the sand barrier was 30 cm, the wind speed acceleration rate was mainly 0.4–0.7. However, when the edge length was 3 m, some areas again reached 0.7–0.8. When comparing the effect of barrier porosity, the wind speed acceleration rate increased with increased porosity, and a higher sand barrier resulted in greater variation in wind speed acceleration rates with porosity. When comparing the effects of the barrier side length, the wind speed acceleration rate increased with an increased side length.

### 3.2. Effective Protection Area

As shown in Figure 5, the wind speed decelerated in the sand barrier grids with different configurations. Thus, when the wind speed was 5 m s^−1^, the wind speed in the sand barrier grids was lower than the threshold wind velocity, and the effective protection area was 100%. Hence, the effective protection area under this wind speed was not compared with that at other wind speeds. The effective protection areas calculated for wind speeds of 8 and 11 m s^−1^ are given in Table 1, which shows that the effective protection area decreased sharply with increased wind speed. When the wind speed was 11 m s^−1^, 23 of the 36 sand barrier configurations did not have effective protection zones, and the effective protection area was more than 10% when the sand barrier had a porosity of 0.2 and a side length of 1 m. When the wind speed was 8m s^−1^, the effective protection area of the sand barriers with different configurations varied greatly and decreased with increased porosity and edge length, and increased with a growth in height.

### 3.3. Roughness

The calculations of the roughness of different configurations of the Salix psammophila sand barrier after wind speed stabilization are shown in Table 2. The roughness of sand barrier grids of different heights varied significantly (p < 0.05) and increased as the barrier height increased. The average roughness of the 30-cm-high sand barrier was 3.01 and 1.69 times that of barriers 10 cm and 20 cm high, respectively. Neither length nor porosity had significant effects on roughness.

### 3.4. Boundary Layer Displacement Thickness

The wind tunnel simulation test showed the wind speed profiles of different configurations of the *Salix psammophila* sand barrier after wind speeds were stable (see Appendix A). The average boundary layer displacement thickness, classified according to barrier specifications, was calculated, as shown in Table 3. The boundary layer displacement thickness increased with the increase in barrier height, and the difference was significant (p < 0.01). The average boundary layer displacement thickness at a height of 30 cm was 2.08 and 1.26 times greater than the values at 10 cm and 20 cm, respectively. The boundary layer displacement thickness varied slightly with length and porosity, but the within-group differences were not statistically significant. However, the numerical values showed a certain regularity, i.e., decreased thickness with increased side length and porosity.

## 4. Discussion

### 4.1. The Boundary Layer Displacement Thickness is More Accurate for Evaluating the Protective Effect of the Sand Barrier

As can be seen from data on the wind speed acceleration rate (Figure 5), the windbreak efficiency of the sand barrier increased with increases in barrier height and decreased with increases in edge length and porosity. As shown in Table 2, the roughness data indicate that windbreak efficiency increased with increased barrier height. However, when evaluating the windbreak effectiveness of sand barriers with different edge lengths and porosities, the results displayed no obvious regularity. The boundary layer displacement thickness data shown in Table 3 indicate that windbreak efficiency increased with a higher barrier height and decreased with a greater length and porosity, which is consistent with the understanding of wind speed acceleration. Therefore, when wind speed acceleration rate was used to measure roughness and boundary layer displacement thickness to evaluate the windproof effectiveness of various configurations of Salix psammophila sand barriers, the results based on boundary layer displacement thickness were more accurate.

To further understand the relationship of roughness and boundary layer displacement thickness to an effective protection area, correlation coefficients were calculated, as shown in Table 4. Roughness and boundary layer displacement thickness were correlated with the effective protection area at the level of p < 0.01. The correlation between roughness and an effective protection area was 0.658, which qualifies as a strong correlation (0.6–0.8), and the correlation between the boundary layer thickness and the effective protection area was 0.811, which shows a very strong correlation (0.8–1.0). This also shows that, compared with roughness, the boundary layer displacement thickness can more accurately reflect the wind speed in the sand barrier grid. Therefore, this, rather than roughness, should be used in the future as the evaluation index.

**Figure 6:**
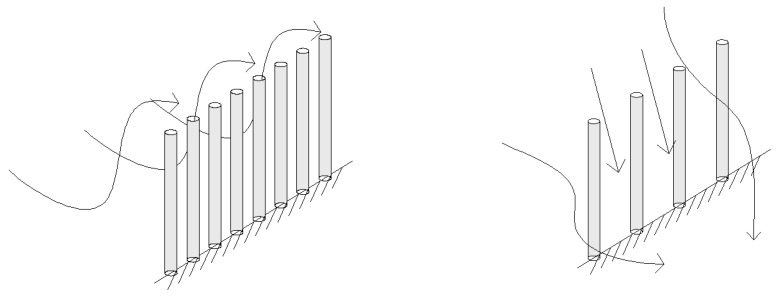
Effect of porosity on airflow.

In previous studies, roughness has been widely used as an important indicator for evaluating the effect of protective measures, and one might ask why it is less accurate when evaluating sand barriers. One possible reason is that the current common height of sand barrier is 10 to 20 cm and the anemometer used for continuous measurement of wind speed in the field is more than 20 cm due to its structure. Thus, the height measured when calculating roughness is often greater than that of the sand barrier. Sand barriers with different porosities have different guiding effects on the airflow. As shown in Figure 6, when the porosity is low, it is difficult for the airflow to pass through the sand barrier. The sand barrier plays an uplifting role in the wind-sand flow, which increases the wind speed above the sand barrier. As currently performed, roughness calculations lead to the conclusion that the protective effect is poor. When porosity is high, the airflow easily passes through the sand barrier, and the uplifting effect of the sand barrier on airflow is not clear. The air flow mainly passes by flowing around, and the wind speed above the sand barrier is lower than the original wind speed because of the energy loss. Thus, the calculation of roughness leads to the conclusion that the protection is better than is actually the case. However, Figure 4 shows that, when the porosity is low, the wind speed acceleration rate is lower, and the sand barrier has a better protection effect. The reason that roughness cannot truly reflect the windproof performance is that the wind speed profile function curve (exponential or logarithmic function) cannot be accurately fitted by wind speed measured only at two heights. The boundary layer displacement thickness can accurately fit the wind speed profile function curve for multiple heights. Therefore, the results can accurately evaluate the effectiveness of wind protection.

### 4.2. Calculation of the Boundary Layer Displacement Thickness

**Figure 7:**
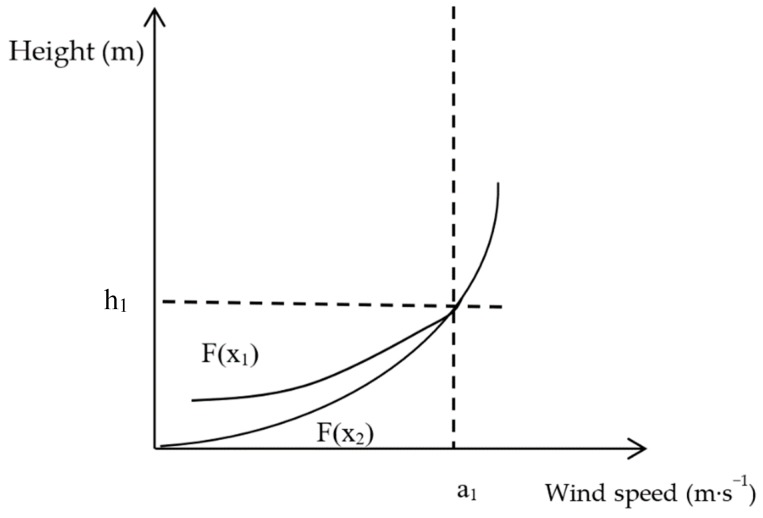
Wind speed profile with or without protective measures (F(x1) that represent the situation with protective measures and F(x2) represents that without protective measures.

In practice, the impact of the underlying surface on the near-surface airflow is large, and the boundary layer displacement thickness is far beyond our measurement range. However, the protective measures have a limited influence on the airflow. As shown in Figure 7, F(x_2_) represents the wind speed profile of bare sandy land without protective measures, and F(x_1_) represents the wind speed profile of sand barriers. When the height is h1, the two function curves intersect, and the speed is a1, which indicates that the sand barrier cannot affect the higher airflow. When the boundary layer displacement thickness is used to evaluate the protective effect of different protective measures, the curve coincidence after a_1_ does not affect the comparison results. Therefore, when evaluating the effect of protective measures, only the integral of the wind speed profile at the effective protection height is required.

**Figure 8:**
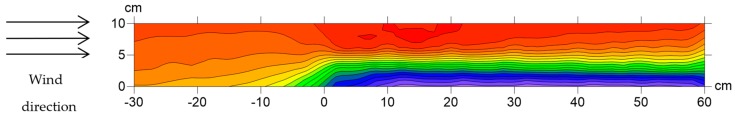
Flow field diagram along a longitudinal section of the wind direction.

Therefore, how do we determine h_1_? Figure 5 shows that sand barriers with small grids, low porosity, and high barriers have the best protective effect. Therefore, we simulated a longitudinal flow field along the wind direction with 0.2 porosity, 1-m length, and 30-cm height, as shown in Figure 8. In this case, the positive coordinate of the abscissa is the area where the sand barrier is laid, the negative coordinate is the area before the barrier, and the wind direction is from the left to the right. It can be seen that, when the airflow reaches the sand barrier, i.e., the 0 coordinate of the abscissa, the contour lines at the height of 0–5 cm are denser and are inclined upward, which indicates that the wind is blocked by the sand barrier and the wind speed decreases. The contour inclines downward in the range of 5 to 10 cm, which indicates that the wind speed above the sand barrier has increased. This is consistent with the results discussed above. When the wind speed reaches a steady state, the contours of different heights tend to be parallel, and the wind speed value in the range of 5 to 10 cm approaches the pre-obstruction wind speed, which indicates that the effective protection height of the sand barrier does not exceed 10 cm. According to the model ratio of 1:10, only a fit of the wind speed profile below 1 m is required to calculate the boundary layer displacement thickness to complete the evaluation of the sand barrier protection effect.

## 5. Conclusions

A wind tunnel simulation test of a *Salix psammophila* sand barrier, which is a typical protective measure in the sandy area of China, was conducted. The surface roughness and boundary layer displacement thickness were calculated to evaluate the windproof effect of the sand barrier at different heights, side lengths, and porosities. The wind speed acceleration rate and effective protection area were measured as evaluation criteria. The results showed that roughness can reflect only the influence of height on the windproof effect of a *Salix psammophila* sand barrier and cannot reflect the influence of side length and porosity. In contrast, the boundary layer displacement thickness can show the influence of multiple factors on the windbreak effect of a *Salix psammophila* sand barrier, and it is strongly correlated with the effective protection area. Based on these findings, when evaluating the effect of protective measures, the boundary layer displacement thickness has wider applicability and accuracy. Therefore, it should replace surface roughness as a new evaluation index.

## Figures and Tables

**Table 1:** Effective protection area (%).

Wind Speed (m·s^−1^)	Porosity	Height (cm)								
		10			20			30		
		Length (m)								
		1	2	3	1	2	3	1	2	3
**8**	0.2	**89.19**	9.68	5.01	89.89	88.82	45.36	**99.79**	**96.89**	**99.83**
	0.3	35.23	0.00	0.00	59.94	71.37	22.00	**99.33**	92.06	72.44
	0.4	15.27	0.00	0.00	41.10	8.75	1.05	**98.08**	72.15	7.55
	0.5	0.00	0.00	0.00	22.76	11.03	0.00	46.08	26.87	0.00
**11**	0.2	**12.64**	0.00	0.00	**13.72**	5.69	6.73	**29.74**	7.37	8.80
	0.3	0.00	0.00	0.00	6.97	0.15	0.00	7.88	1.86	0.00
	0.4	0.00	0.00	0.00	0.00	0.00	0.00	5.19	2.12	0.00
	0.5	0.00	0.00	0.00	0.00	0.00	0.00	0.00	0.00	0.00

**Table 2:** Roughness of *Salix psammophila* sand barriers with different configurations: cm.

Length/m	Height/m	Porosity			
		0.2	0.3	0.4	0.5
**1**	0.1	0.0145 ± 0.0151a	0.0202 ± 0.0067a	0.0111 ± 0.0018a	0.0022 ± 0.0010a
	0.2	0.0348 ± 0.0170b	0.0508 ± 0.0025b	0.0433 ± 0.0070b	0.0232 ± 0.0137b
	0.3	0.0640 ± 0.0258c	0.0815 ± 0.0249c	0.0526 ± 0.0264c	0.0311 ± 0.0150c
**2**	0.1	0.0343 ± 0.0065a	0.0417 ± 0.0265a	0.0193 ± 0.0057a	0.0173 ± 0.0111a
	0.2	0.0447 ± 0.0122b	0.0425 ± 0.0130b	0.0228 ± 0.0187b	0.0197 ± 0.0541b
	0.3	0.0603 ± 0.0112c	0.0620 ± 0.0300c	0.0517 ± 0.0204c	0.0602 ± 0.0330c
**3**	0.1	0.0117 ± 0.0087a	0.0118 ± 0.0012a	0.0136 ± 0.0134a	0.0081 ± 0.0035a
	0.2	0.0225 ± 0.0058b	0.0204 ± 0.0099b	0.0197 ± 0.0032b	0.0219 ± 0.0102b
	0.3	0.0332 ± 0.0204c	0.0377 ± 0.0030c	0.0544 ± 0.0116c	0.0310 ± 0.0038c

Different letters represent significant differences (*p* < 0.05) among the heights. Data represent mean ±standard deviation (SD).

**Table 3:** Boundary layer displacement thickness of *Salix psammophila* barriers with different specifications.

Length/m	Height/m	Porosity			
		0.2	0.3	0.4	0.5
**1**	0.1	1.83 ± 0.11A	1.87 ± 0.02A	1.66 ± 0.04A	1.41 ± 0.09A
	0.2	3.00 ± 0.03B	2.87 ± 0.08B	2.72 ± 0.08B	2.44 ± 0.13B
	0.3	3.64 ± 0.10C	3.59 ± 0.05C	3.51 ± 0.01C	3.22 ± 0.16C
**2**	0.1	1.82 ± 0.07A	1.71 ± 0.14A	1.58 ± 0.04A	1.54 ± 0.03A
	0.2	2.99 ± 0.12B	2.79 ± 0.09B	2.62 ± 0.17B	2.32 ± 0.18B
	0.3	3.40 ± 0.08C	3.45 ± 0.18C	3.27 ± 0.16C	3.09 ± 0.18C
**3**	0.1	1.58 ± 0.14A	1.40 ± 0.16A	1.35 ± 0.16A	1.36 ± 0.05A
	0.2	2.62 ± 0.06B	2.55 ± 0.07B	2.52 ± 0.07B	2.19 ± 0.17B
	0.3	3.55 ± 0.20C	3.39 ± 0.08C	3.07 ± 0.12C	2.64 ± 0.05C

Different letters represent highly significant differences (*p* < 0.01) among heights. Data represent mean ± standard deviation (SD).

**Table 4:** Correlations of roughness and the boundary layer displacement thickness with an effective protection area.

Measure Index	Parameter	Roughness	Displacement Thickness of the Boundary Layer
Effective protection area	Correlation Coefficient	0.658 **	0.811 **
	Significant (2-tailed)	0.000	0.000
	N	72	72

** Correlation is significant at the 0.01 level (2-tailed).

## Appendix A

**Table:**

Length/m	Height/m	Porosity	Speed m/s	A	B	R^2^	D
1	0.1	0.2	5	0.0761	0.9181	0.9429	0.7687
			8	0.0879	0.5597	0.9676	1.8581
			11	0.0823	0.4155	0.9876	1.9321
		0.3	5	0.0738	0.9070	0.9856	1.8770
			8	0.0713	0.5823	0.9882	1.8815
			11	0.0785	0.4165	0.9725	1.8351
		0.4	5	0.0397	1.0623	0.9892	1.6877
			8	0.0441	0.6614	0.9818	1.6827
			11	0.0292	0.5110	0.9918	1.6160
		0.5	5	0.0210	1.1834	0.9778	1.4652
			8	0.0187	0.7455	0.9806	1.4496
			11	0.0096	0.5961	0.9801	1.3005
	0.2	0.2	5	0.6488	0.4893	0.9754	3.0127
			8	0.6346	0.3091	0.9795	3.0216
			11	0.5781	0.2345	0.9806	2.9650
		0.3	5	0.5499	0.5198	0.9839	2.9490
			8	0.5099	0.3342	0.9832	2.8812
			11	0.4579	0.2528	0.9837	2.7905
		0.4	5	0.4656	0.5370	0.9826	2.7996
			8	0.4505	0.3468	0.9763	2.7131
			11	0.4242	0.2528	0.9693	2.6515
		0.5	5	0.2405	0.7049	0.9881	2.4587
			8	0.2612	0.4232	0.9869	2.4346
			11	0.2328	0.3148	0.9830	2.3262
	0.3	0.2	5	0.7896	0.4904	0.9980	3.6937
			8	0.7903	0.3038	0.9974	3.7029
			11	0.7365	0.2208	0.9998	3.5277
		0.3	5	0.8013	0.4637	0.9910	3.6404
			8	0.7755	0.2922	0.9977	3.5497
			11	0.7738	0.2170	0.9926	3.5876
		0.4	5	0.7448	0.4798	0.9928	3.5243
			8	0.7126	0.3030	0.9958	3.5030
			11	0.7420	0.2195	0.9878	3.5164
		0.5	5	0.6346	0.5312	0.9828	3.3828
			8	0.6001	0.3334	0.9976	3.2343
			11	0.5465	0.2449	0.9908	3.0602
2	0.1	0.2	5	0.0571	0.9944	0.9959	1.8568
			8	0.0347	0.7152	0.9996	1.7400
			11	0.0519	0.4587	0.9993	1.8633
		0.3	5	0.0296	1.1083	0.9976	1.7545
			8	0.0353	0.6884	0.9919	1.8341
			11	0.0178	0.5496	0.9995	1.5552
		0.4	5	0.0206	1.2185	0.9999	1.6037
			8	0.0164	0.7724	0.9976	1.5951
			11	0.0152	0.5772	0.9999	1.5295
		0.5	5	0.0186	1.1681	0.9983	1.5530
			8	0.0152	0.7854	0.9984	1.5062
			11	0.0135	0.5747	0.9909	1.5735
	0.2	0.2	5	0.5551	0.5441	0.9848	3.0665
			8	0.4373	0.3843	0.9999	3.0351
			11	0.4009	0.2725	0.9887	2.8486
		0.3	5	0.3815	0.6226	0.9881	2.7805
			8	0.3630	0.4028	0.9976	2.8786
			11	0.3236	0.2958	0.9887	2.7122
		0.4	5	0.2730	0.6839	0.9947	2.8072
			8	0.2649	0.4230	0.9935	2.5777
			11	0.2125	0.3376	0.9903	2.4653
		0.5	5	0.2358	0.7176	0.9989	2.5606
			8	0.1543	0.4991	0.9982	2.4177
			11	0.1213	0.3880	0.9995	2.2381
	0.3	0.2	5	0.7337	0.5064	0.9905	3.4385
			8	0.7298	0.3167	0.9927	3.4604
			11	0.6490	0.2383	0.9982	3.3139
		0.3	5	0.6907	0.5201	0.9958	3.6276
			8	0.6477	0.3344	0.9969	3.4376
			11	0.6086	0.2476	0.9953	3.2709
		0.4	5	0.5727	0.5765	0.9894	3.4474
			8	0.5505	0.3564	0.9998	3.2297
			11	0.5165	0.2600	0.9970	3.1313
		0.5	5	0.4166	0.6469	0.9825	3.2634
			8	0.3940	0.4000	0.9873	3.1085
			11	0.3323	0.3041	0.9980	2.9147
3	0.1	0.2	5	0.0092	1.3559	0.9880	1.4524
			8	0.0250	0.7307	0.9862	1.7169
			11	0.0149	0.5741	0.9796	1.5584
		0.3	5	0.0131	1.2864	0.9563	1.5047
			8	0.0116	0.8108	0.9637	1.4812
			11	0.0026	0.7333	0.9583	1.2134
		0.4	5	0.0108	1.3634	0.9860	1.5320
			8	0.0054	0.9247	0.9992	1.3108
			11	0.0017	0.7852	0.9759	1.2059
		0.5	5	0.0060	1.3867	0.9999	1.3475
			8	0.0066	0.8912	0.9963	1.4102
			11	0.0045	0.6649	0.9996	1.3121
	0.2	0.2	5	0.2816	0.7130	0.9638	2.6837
			8	0.2071	0.4989	0.9922	2.6226
			11	0.1963	0.3570	0.9852	2.5561
		0.3	5	0.1792	0.8008	0.9960	2.5148
			8	0.2199	0.4695	0.9942	2.6268
			11	0.2335	0.3306	0.9905	2.5071
		0.4	5	0.2417	0.7093	0.9954	2.5957
			8	0.1751	0.4963	0.9993	2.4756
			11	0.1770	0.3543	0.9911	2.4776
		0.5	5	0.1131	0.8835	0.9970	2.2952
			8	0.1214	0.5314	0.9946	2.1115
			11	0.0794	0.4211	0.9961	2.0010
	0.3	0.2	5	0.9478	0.4565	0.9364	3.7694
			8	0.8657	0.2949	0.9472	3.4952
			11	0.6275	0.2550	0.9872	3.3870
		0.3	5	0.6217	0.5652	0.9938	3.4790
			8	0.5293	0.3687	0.9878	3.3525
			11	0.5122	0.2721	0.9872	3.3260
		0.4	5	0.3514	0.7024	0.9985	2.9311
			8	0.4373	0.3959	0.9955	3.1399
			11	0.4193	0.2916	0.9857	3.1540
		0.5	5	0.2367	0.7455	0.9957	2.6805
			8	0.2427	0.4611	0.9997	2.6475
			11	0.1886	0.3706	0.9835	2.5904

The fitting function is D = A*e*^Bx^.
